# Unravelling drought and salinity stress responses in barley genotypes: physiological, biochemical, and molecular insights

**DOI:** 10.3389/fpls.2024.1417021

**Published:** 2024-07-10

**Authors:** Hameed Alsamadany, Abdulbaki Shehu Abdulbaki, Yahya Alzahrani

**Affiliations:** ^1^ Department of Biological Sciences, Faculty of Science, King Abdulaziz University, Jeddah, Saudi Arabia; ^2^ Department of Plant Science and Biotechnology, Faculty of Life Sciences, Federal University Dutsinma, Katsina, Nigeria

**Keywords:** abiotic stress, barley, genotype-specific responses, chlorophyll content, stomatal conductance, antioxidant enzyme activities

## Abstract

In the face of escalating environmental challenges, understanding crop responses to abiotic stress is pivotal for sustainable agriculture. The present study meticulously investigates the intricate interplay between drought and salinity stress in barley (*Hordeum vulgare* L.). Employing three distinct barley genotypes—Traveller, Prunella, and Zahna—we scrutinize their physiological, biochemical, and molecular adaptations under stress conditions. Our findings underscore genotype-specific responses, unravelling the multifaceted mechanisms that govern stress tolerance. Chlorophyll content, a vital indicator of photosynthetic efficiency, exhibits significant variations across genotypes. Salinity stress induces a decline in chlorophyll levels, while drought stress triggers a more nuanced response. Stomatal conductance, a key regulator of water loss, also diverges among the genotypes. Traveller displays remarkable stomatal closure under drought, conserving water, whereas Prunella and Zahna exhibit contrasting patterns. Antioxidant enzyme activities, crucial for combating oxidative stress, fluctuate significantly. Activities of superoxide dismutase (SOD) and catalase (CAT) surge under salinity stress, while drought predominantly impacts SOD. Gene expression profiling reveals genotype-specific signatures, with stress-responsive genes modulating adaptive pathways. Correlation analyses revealed the intricate interplay of the physiological and biochemical parameters. Genotype-specific adaptations, coupled with dynamic physiological and molecular responses, underscore the plasticity of barley’s stress tolerance mechanisms. Throughout the study, the Zahna genotype demonstrated notable tolerance in terms of performance. These insights hold promise for breeding resilient cultivars, bolstering food security in an increasingly unpredictable climate. By deciphering the barley stress symphony, we contribute to the harmonious orchestration of sustainable agricultural practices.

## 1 Introduction

Barley is a cereal plant species belonging to the Triticeae tribe, being cultivated all over the world. Barley has a very flexible growth regime; it is often grown in a multitude of environments with considerable variability in reliable yields, particularly in the presence of adverse conditions (Dhima et al., 2021; Tesfaye and Bayih, 2024). The crop has diverse uses, such as in the food and feed, pharmaceutical, and malting industries (Geng et al., 2022). Barley rates as the fourth most crucial cereal on a global scale in both area of cultivation and in quantity produced (Badea and Wijekoon, 2021).

Abiotic stresses such as drought and salinity are the most serious factors affecting crop production, limiting crop yields worldwide (Ma et al., 2020). Barley crop exhibits greater tolerance to abiotic stresses by comparison with other common grains such as rice and wheat, allowing for cultivation in various environments (Zinati et al., 2021; Akbari et al., 2022). Notwithstanding, drought can significantly impacts barley yield, growth, and development in several countries in the world (Li et al., 2020). Drought stress leads to serious damages to physiological parameters such as photosynthetic apparatus (Ghotbi-Ravandi et al., 2021), biochemical traits such as accumulation of osmolytes and agronomic characteristics such as grain development impairment and shorter grain filling duration in barley (Hassan et al., 2019; Hong and Zhang, 2020). Salinity stress also affects barley growth negatively, with studies showing a decline in physiological parameters such as chlorophyll concentration and stomatal conductance (Narimani et al., 2020; Tobiasz-Salach et al., 2021). Factors like Na+ exclusion and osmotic imbalance are associated with salt-stressed barley (Alkharabsheh et al., 2021; Fu et al., 2022). Simultaneously-occurring drought and salinity exacerbate the menace caused by the individual stresses both physiologically and biochemically (Angon et al., 2022). Drought and salinity stresses often occur during the early growth stages of plants, which are critical for determining their final yield and quality. Therefore, understanding the responses of plants to abiotic stress at this stage is essential for developing effective strategies to enhance crop resilience and adaptation (Tarnawa et al., 2023).

Thus, the abiotic stress tolerance in plants is critical for their survival and productivity. Mitigating abiotic stress tolerance in plants involves various strategies to help them cope with and adapt to challenging environmental conditions. One approach is to improve soil quality through practices such as adding organic matter, using cover crops, and implementing proper irrigation techniques to enhance water retention and nutrient availability (Imran et al., 2022). Another strategy is to select and breed crop varieties that exhibit higher tolerance to specific stress factors, such as drought or salinity. Moreover, several studies have identified genes, transcription factors, and QTLs connected to tolerance to drought in barley (Harb and Samarah, 2015; Hasanuzzaman et al., 2019). Genetic variations and proteomic analysis have helped identify QTLs and proteins related to salt tolerance in barley genotypes (Zhu et al., 2020; Mariey et al., 2023). For example, genes like HvDREB3, HvDHN3, and HvSOD, HKT1;5 have been identified for their established roles in drought and salinity stress responses, including transcriptional regulation, osmoprotection, antioxidant defense, and ion homeostasis (Gruszka et al., 2020; Uçarlı and Gürel, 2020; Kumar et al., 2023).

The present research aims to characterize the biochemical and physiological changes occurring at the molecular level in barley plants subjected to salinity and drought stress conditions at the seedling stage where plants are most vulnerable. This includes unravelling the detrimental roles played by these stresses on barley physiological and biochemical process. Additionally, the study seeks to elucidate the dynamics of gene expression and protein synthesis associated with stress adaptation and tolerance mechanisms in barley plants, shedding light on the intricate interplay between different stress signaling pathways and the integration of stress responses in the face of simultaneous salinity and drought stresses. Ultimately, these insights contribute to the development of sustainable agricultural practices and crop management strategies aimed at enhancing barley productivity in regions susceptible to salinity and drought stress.

## 2 Materials and methods

### 2.1 Description of the barley cultivars and treatment application

The seeds of the barley cultivars used in the current investigation were collected from the Lake Chad Research Institute (LCRI), Nigeria. The cultivars are namely Traveller, Prunella and Zahna. The experiment was conducted using 20 kg size porcelain pots (with a 30 cm diameter) filled with 15kg peat moss and soil at the experimental area of the Department of Biological Sciences, King Abdulaziz University, Jeddah, Saudi Arabia.

The study was developed to evaluate barley plants morphologically, physiologically, biochemically and molecularly under combined and individual treatments of drought and salinity stresses using three factorial arrangements in a randomized complete block design (RCBD) with three replications. In this case, 3 x 9 x 3 factorial arrangement was applied representing genotypes, treatment/stress conditions and replications respectively. The treatments details include C-control, D1- 10 days withholding irrigation, D2- 20 days withholding irrigation, S1- 200mM NaCl, S2- 300mM NaCl, D1S1- 10days+200mM NaCl, D1S2- 10days+300mM NaCl, D2S1- 20days+200mM NaCl, and D2S2- 20days+300mM NaCl. Stress imposition was done at the seedling stage. Salinity stress was created by application of 100 mM and 200 mM NaCl at weekly intervals. Measurements were taken on a weekly basis following the stress imposition.

### 2.2 Collection of data

#### 2.2.1 Physiological qualities

To determine the chlorophyll content of the leaves, the method described by Mahmood et al. (2016) was used. Essentially, chlorophyll was obtained by soaking 0.5 g of fresh leaf samples in a shaker with 80% acetone until the leaves lost their color. The resulting extract was then centrifuged at 13,000 rpm for 10 minutes, and the liquid above the sediment was utilized to determine the total chlorophyll (both a and b) levels at 663 and 645nm absorbance with a spectrophotometer.

The CIRAS-3 equipment (updated version SC-1 (Amesbury, MA 01913, USA) was employed to spontaneously assess the stomatal conductance (gs) (μmol H_2_O m^−2^ s^−1^) and photosynthesis rate (pn) by placing it on fully expanded leaves.

The methodology described by Alghabari et al. (2021) was utilized for measuring the cell membrane stability. This was done by using leaf pieces (100 mg) placed in two separate tubes, each containing (20 mL) deionized water. A tube was then incubated for 30 minutes at 40°C to measure its conductivity (C1), while the other tube was maintained at 100°C for 10 minutes to measure its conductivity (C2).

Finally, the percentage difference was calculated using the formula:


$$1-\left(\frac{C1}{C2}\right)*100$$

The estimation of the leaf relative water content (RWC) was as thus:


$$\text{Relative Water Content}=\frac{Wf-Wd}{Wt-Wd}\times 100$$

Wf=fresh weight, Wt=turgid weight, Wd=dry weight

Water and solute potentials were measured using the method adopted by Sattar et al. (2020). Na/K content was analyzed from the leaves in accordance with the method of Hniličková et al. (2019).

#### 2.2.2 Biochemical qualities

Activities of antioxidant enzymes namely catalase (CAT), superoxide dismutase (SOD) and peroxidase (POD) were assessed using commercially available kits. CAT activity was measured in accordance with the protocol provided by the manufacturer of the catalase analytical kit (Product No. MBS8243260, MyBioSource, United States). SOD activity was determined using the SOD1 ELISA kit (Product No. MBS283325, MyBioSource, United States) as per the manufacturer’s instructions. Additionally, POD activity was evaluated using the peroxidase activity analytical kit (Product No. E-BC-K227-S, Elabscience, United States) accordingly with the specified protocol. The activities of the antioxidant enzymes were quantified as units per milligram of protein (Umg^-1^) of protein using their individual standard absorbance curves.

Similar spectrophotometric method was opted for in measuring the glycine betaine based on its reaction with iodine as described by Valadez-Bustos et al. (2016). Proline was assayed for in the same pattern of its reactivity with ninhydrin while utilizing a UV–vis spectrophotometer (DeNovix, United States, Product No. DS 11FX).

The protocol for assessing lipid peroxidation using MDA content involves the reaction of malondialdehyde (MDA) content with thiobarbituric acid (TBA) to form a pink chromogen that can be measured spectrophotometrically.

The amount of MDA in the sample is calculated from a standard curve and interpreted as nmol/mg protein (Reilly and Aust, 1999). The content of the superoxide anion radical (O_2_^−^) was assessed using the method outlined by Ajiboye et al. (2016). Additionally, the estimation of hydrogen peroxide (H_2_O_2_) concentration was based on the approach outlined by Velikova et al. (2000).

#### 2.2.3 Morphological qualities

From randomly selected plants, plant height and leaf qualities including number of leaves and leaf area was collated manually.

#### 2.2.4 Molecular qualities

The RNA was isolated from chosen plants utilizing the Qiagen RNeasy kit (Qiagen, United States) following the procedure outlined by Li et al. (2018). Subsequently, adhering to the same protocol, a cDNA library was generated, utilizing 2 µg of RNA as specified by the manufacturer. Similarly, the SYBR Green 1 master kit was employed for qRT-PCR analysis as per the manufacturer’s guidelines. Additionally, gene expression was standardized using the Actin-expressing gene (Vradi03g00210). **Table 1** shows the list of primers used.

**Table 1:** List of primers utilized for qRT-PCR analysis.

Primers	Forward	Reverse
*HvDREB3*	CAGAACCACTGGCTCCACCTC	ACGCTGCGGCAAAAGACGTCG
*HvDHN3*	GTGATCAGCAGCAGACCGG	CATGATGCCCTTCTTCTCGC
*HvCAT2*	TGCAGGAGTACTGGCGTCTTCGACTT	AGATCCCGGGCACGAGGCCGGGGCC
*HvSOD*	ATGGTGAAGGCTGTTGCTGTGC	TCAGCCTTGAAGTCCGATGATCCC
*HvAPX*	GGAGTTGTCGCCGTGGAGGTGTCCGGTG	CAAGATCACCCTGGTCGCGCATAGTAGC
*HvPRP1*	AAGACACACTAGCTCGACTTC	CCACTGCCGCATGAGACGTCG
*HvHKT1;5*	GCAGATCTCCGATGACCCAC	TGAGCCTGCCGTAGAACATG
*HvACTIN*	CGTGTTGGATTCTGGTGATG	AGCCACATATGCGAGCTTCT

### 2.3 Statistical analysis

The Statistix 8.1 software was utilized to conduct the analysis of variance (ANOVA) at a significance level of 5%. Additionally, for the principal component analysis (PCA), correlation and heatmap analyses, RStudio version 1.3.959 was utilized.

## 3 Results

### 3.1 Physiological qualities

**Figure 1:**
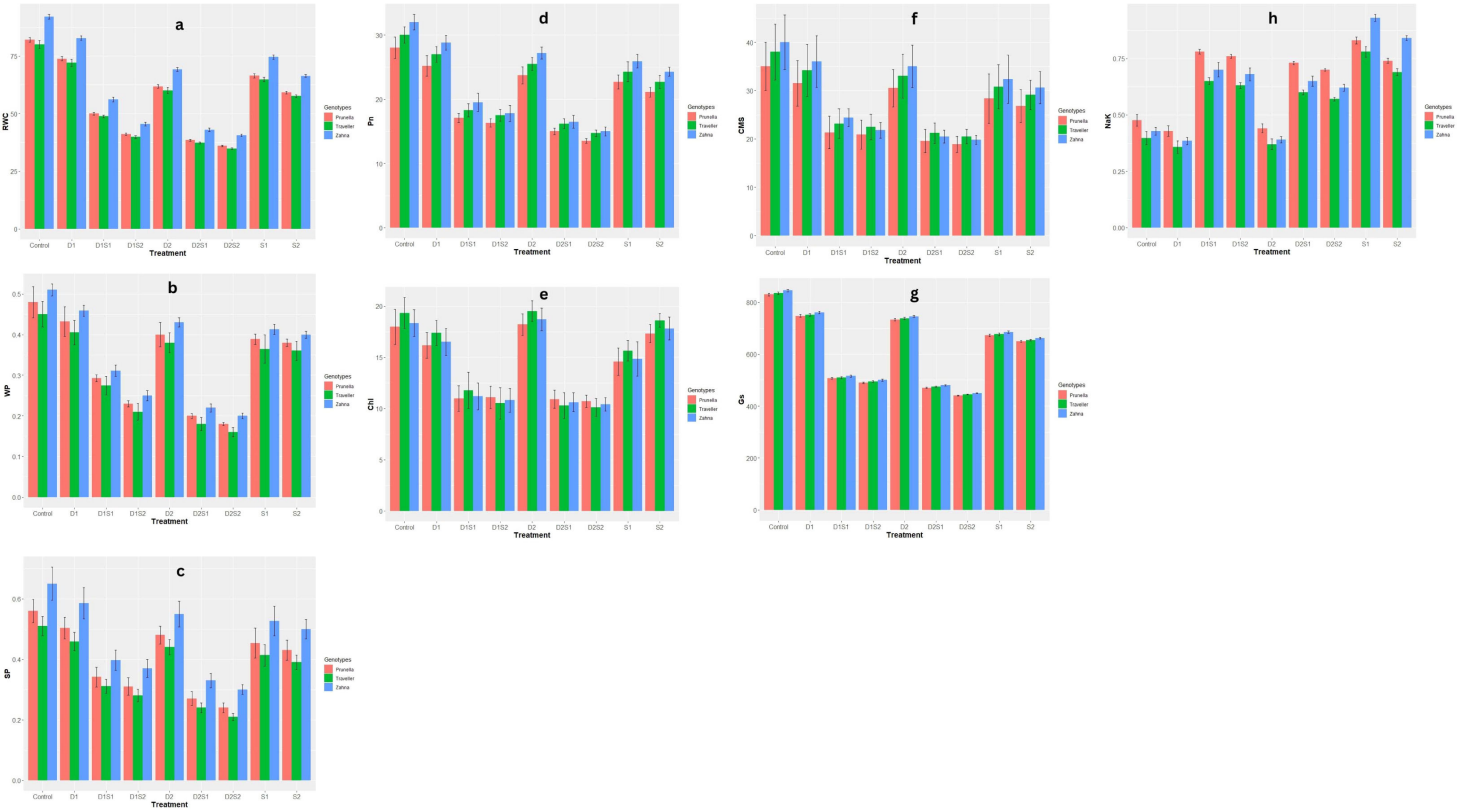
Effect of individual and combined treatments of drought and salinity on physiological qualities of barley genotypes. **(A)** RWC, relative water content; **(B)** WP, water potential; **(C)** SP, solute potential; **(D)** Pn, photosynthesis rate; **(E)** Chl, Chlorophyll content; **(F)** CMS, Cell membrane stability; **(G)** Gs, stomatal conductance; **(H)** NaK, Na^+^/K^+^ ratio. Vertical bars are mean with error bars at p≤ 0.05.

The individual treatments of drought and salinity (D1, D2, S1, S2) also show relatively high Chlorophyll and CMS values (**Figures 1E, F**), suggesting these conditions are less stressful or more favorable for maintaining higher Chlorophyll levels compared to the combined treatments. Generally, While the genotype Traveller tends to show higher Chlorophyll values compared to Zahna and Prunella, Zahna shows higher CMS values across most treatments. Zahna also appears to be more resilient across most treatments, maintaining higher RWC levels, and water and solute potentials (**Figures 1A–C**). Under treatment conditions, there is a general trend of reduced RWC, Sp and Wp compared to the control, with the most reduction observed with the integrated drought and salinity treatments. Also, for Photosynthetic rate (Pn) levels (**Figure 1D**), Zahna consistently exhibits a relatively higher tolerance to both drought and salinity, maintaining higher photosynthetic rate compared to Prunella and Traveller throughout the treatments. The genotypes, however, demonstrated similar stomatal conductance (Gs) values across all treatments (**Figure 1G**). Notwithstanding, a noticeable decline in the Gs values is recorded in all the treatments, especially in the (combined treatments) compared to the control. All the treatments induced an increase in Na/K levels across all genotypes (**Figure 1H**). The increase was most pronounced in Prunella, except in the sole salinity treatments (S1, S2), where it was more pronounced in Zahna. Comparing the parameters, it is interesting to note that aside the Na/K, where salinity had higher values than drought, drought had better values, in terms of stress tolerance, in all other parameters. This suggests that the barley genotypes are more susceptible to salinity than drought.

### 3.2 Biochemical qualities

**Figure 2:**
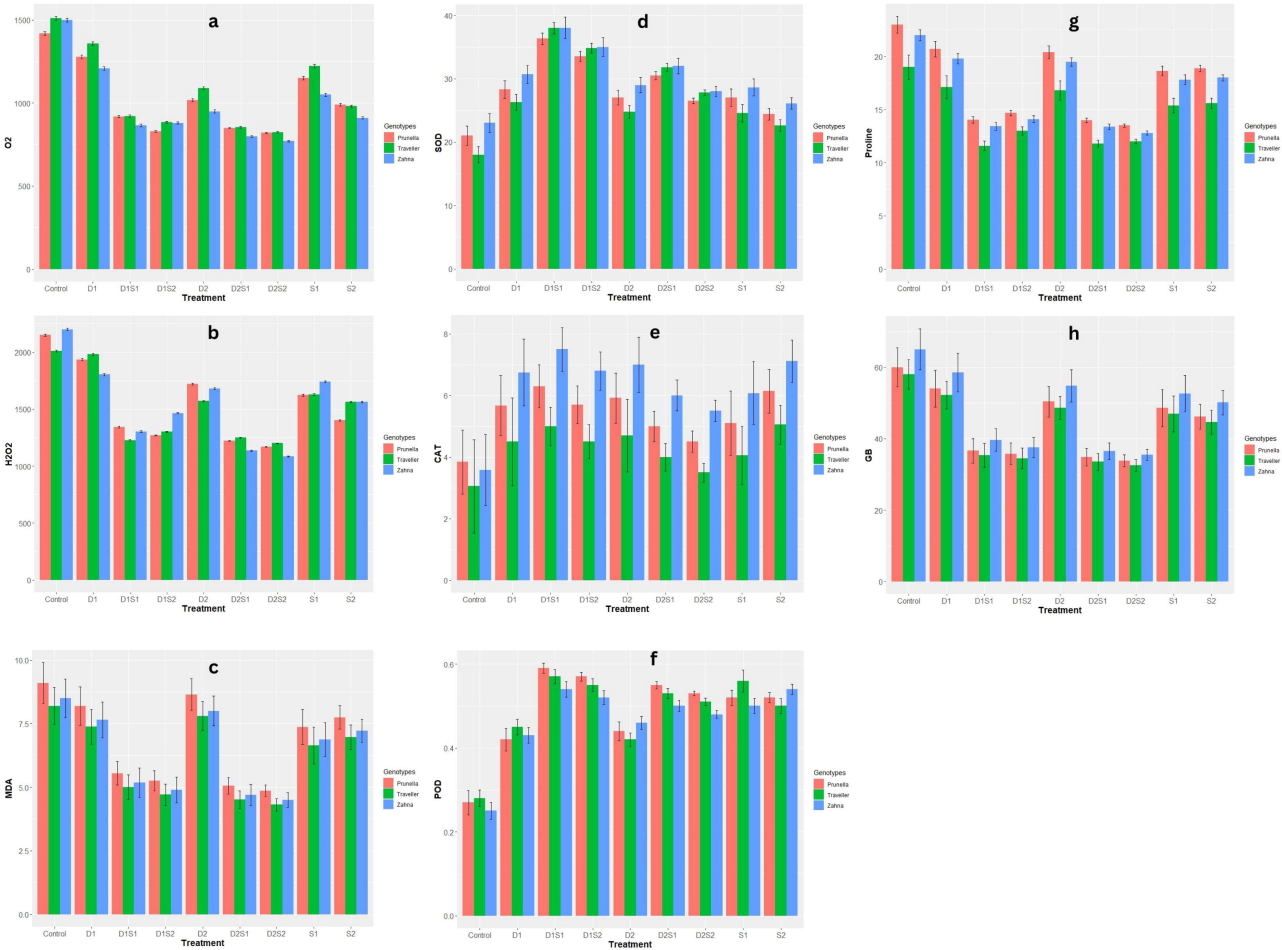
Effect of individual and combined treatments of drought and salinity on biochemical qualities of barley genotypes. **(A)** O_2_-, Superoxide anion radical; **(B)** H_2_0_2_, Hydrogen peroxide; **(C)** MDA, Malondialdehyde; **(D)** SOD, superoxide dismutase; **(E)** CAT, catalase; **(F)** POD, peroxidase; **(G)** Proline; **(H)** GB, glycine betaine. Vertical bars are mean with error bars at p≤ 0.05.

Among the genotypes, varying levels of H_2_O_2_ concentration were noticed (**Figure 2B**). Prunella had higher H_2_O_2_ levels in certain treatments (D2, D1S1), Zahna showed higher H_2_O_2_ levels in other treatments (S1, D1S2), and Traveller had the highest H_2_O_2_ levels in others (D1, D2S1). Both levels of drought and salinity conditions (D1, D2, S1 and S2) show a decrease in H_2_O_2_ production compared to the control across all genotypes. A further noticeable decline in H_2_O_2_ levels was recorded in the combination treatments compared to single stress treatments. Zahna had lower superoxide (O_2_^-^) levels in most treatments compared to Traveller and Prunella, with similar superoxide (O_2_^-^) levels. Both individual droughts and salinity stress reduced O_2_^-^levels in all genotypes relative to the control (**Figure 2A**). The reduction was slightly more severe in salinity than under drought conditions. However, the combined treatments (D1S1, D1S2, D2S1, and D2S2) generally resulted in the lowest O_2_^-^levels. Also, especially in the combined stress, the malondialdehyde (MDA) concentration was reduced in all the treatments when compared with the control with Prunella having a relatively higher concentration among the genotypes in all treatments and control (**Figure 2C**).

When subjected to individual drought and salinity, an increase in SOD activity was observed across all genotypes (**Figure 2D**). The combined stress of drought and salinity (especially D1S1 and D1S2) triggers a further increase in SOD activity in all genotypes. Zahna demonstrates remarkable resilience, maintaining stable levels of SOD in most treatments. Compared with the control. All stress conditions also amplified the CAT levels with Zahna followed by Prunella, maintaining higher CAT levels under all stress conditions (**Figure 2E**). Different from the severe drought and sole salinity treatments (D2, S1, and S2), Prunella exhibited higher POD in all treatments (**Figure 2F**). All stress conditions, however, elevated the POD activity.

The stress conditions led to a decline in proline and glycine betaine (GB) levels, with most decline observed in the integrated drought and salinity (**Figures 2G, H**). Prunella and Zahna accounted for higher proline and glycine betaine levels compared to Traveller.

### 3.3 Morphological qualities

Results revealed notable variations in the morphological parameters, including plant height (PH), number of leaves (NL), leaf length (LL), leaf breadth (LB), and leaf area (LA) across different treatments and genotypes (**Table 2**). Salinity treatment (S) tends to promote plant growth (higher PH, NL, and LA), while drought treatment (D) has a mixed effect. Treatments such as D2S1 consistently produced taller plants (62.03 cm) and larger leaf areas (78.15 cm²), while S2 recorded the highest number of leaves (6.16 leaves). Among genotypes, Zahna generally exhibited taller plants with more leaves, albeit with shorter and narrower leaves compared to other genotypes like Prunella and Traveller. The interaction effect between genotype and treatment is significant for all traits (PH, NL, LL, LB, LA) based on the p-values (all< 0.05).

**Table 2:** Effect of individual and combined treatments of drought and salinity on Morphological characteristics of barley genotypes.

Treatment (T)	PH	NL	LL	LB	LA
C	52.59c	5.33bc	41.83cd	1.40ab	72.80a
D1	52.61c	5.77ab	38.56d	1.16c	50.56b
D1S1	60.49ab	6.00ab	48.38a	1.36abc	72.21a
D1S2	59.55ab	6.00ab	46.00abc	1.40ab	72.83a
D2	54.98bc	5.61ab	42.01bcd	1.34bc	65.92ab
D2S1	62.03a	5.61ab	46.71ab	1.56a	78.15a
D2S2	51.43c	4.72c	38.87d	1.20bc	50.29b
S1	56.48abc	5.50ab	41.45cd	1.57a	70.24a
S2	60.14ab	6.16a	44.87abc	1.31bc	63.50ab
Genotypes (G)	PH	NL	LL	LB	LA
ZAHNA	46.59b	5.77a	32.77c	0.82b	27.06c
PRUNELLA	45.50b	5.62a	30.87c	0.82b	26.31c
TRAVELLER	44.71b	5.33a	39.51b	0.76b	31.79c
G	0.0001	0.34	0.0001	0.0001	0.0001
T	0.0001	0.001	0.0001	0.0001	0.002
G*T	0.0001	0.20	0.0001	0.0001	0.0001

Values with the different lowercase letters are significantly different at p≤0.05.* represents interactions.

### 3.4 Gene expression studies

**Figure 3:**
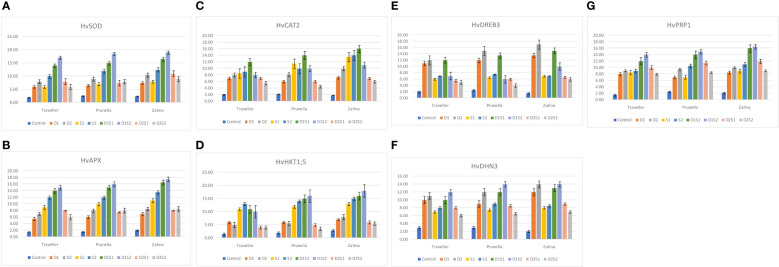
Relative expression of drought and salinity-related genes in different barley genotypes during individual and combined application of drought and salinity stresses. **(A)** HvSOD, **(B)** HvAPX, **(C)** HvCAT2, **(D)** HvHKT1;5, **(E)** HvDREB3, **(F)** HvDHN3, **(G)** HvPRP1.

Gene expression results are presented in (**Figures 3A–G**) Across genotypes, all the treatments demonstrated similar expression patterns of HvSOD, HvAPX and HvCAT. While the treatment D1S2 (moderate drought with severe salinity) had the highest expression of HvSOD and HvAPX, D1S1 (moderate drought with moderate salinity) had the greatest expression of HvCAT2 in all genotypes. Taken together, the combination treatments involving moderate drought (D1) have the highest expression in enzymatic antioxidant-related genes. Generally, Zahna showed slightly stronger HvCAT2 and HvAPX activities compared to Traveller and Prunella under most treatments. Furthermore, even though the expression pattern of HvHKT1;5 and HvPRP1 was similar in most treatments across the genotypes, the highest expression of the two genes was found in Zahna under the treatment D1S2 and in D2 for HvDREB3. For HvDHN3, the highest upregulation was shown in both Prunella and Zahna under the D1S2 treatment and equally in Zahna in the D2 treatment.

### 3.5 Correlation analysis

**Figure 4:**
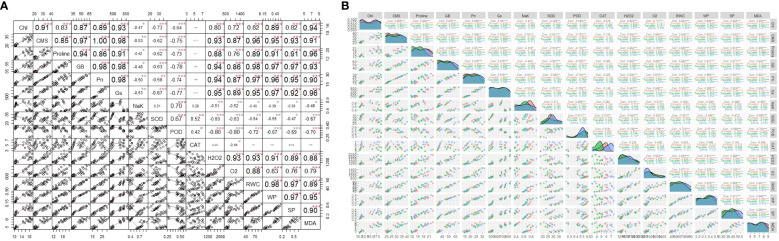
**(A)** The correlations among factors. The upper matrix shows the Pearson coefficients, and results were significant at *** p < 0.01, ** p < 0.05, or * p < 0.1 as marked. The red solid lines in the lower matrix show a smooth regression between the two factors. **(B)** Pearson correlation matrixes of both individual and combined drought and salinity stresses effects on barley genotypes (* Significant at α 0.1, ** significant at α 0.01, *** significant at α 0.001).

Some of the physiological traits revealed strong positive correlation with each other and with some of the biochemical traits that indicate stress response (**Figures 4A, B**). For instance, the physiological traits (RWC, WP, SP) are highly positively correlated with each other and negatively correlated with the antioxidant enzymes (SOD, POD). However, negative correlation is observed between some physiological parameters like NaK and CMS or Chl. Also, non-negligible positive correlation existed between certain physiological and biochemical parameters like Chl and proline.

**Figure 5:**
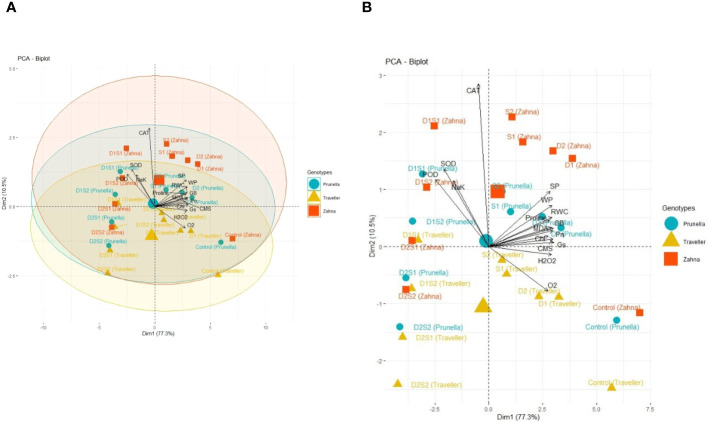
**(A)** PCA scatter plot illustrating the clustering of physiological and biochemical parameters based on their similarity and dissimilarity, specifically in relation to different barley cultivars. **(B)** PCA scatter plot illustrating the clustering of physiological and biochemical parameters based on their similarity and dissimilarity. These parameters were influenced by both individual drought and salinity treatments, as well as their combined effects.

Based on the PCA biplots (**Figures 5A, B**), Prunella and Traveller are observed more similar to each other than to Zahna in terms of their biochemical responses to the treatments, as they are closer to each other on the biplots and have smaller ellipses. Zahna is more resistant to the treatments than Prunella and Traveller, as it shows more variation and dispersion on the biplots and has larger ellipses. Traveller samples show more association with the ROS (H_2_O_2_ and O_2_) compared to other genotypes. The control treatment has the least effect on the biochemical parameters, as it is close to the origin of the biplots and has small arrows pointing to it.

The D1S1 treatment has the most effect on the biochemical parameters, as it is far from the origin of the biplots and has large arrows pointing to it. Many of the treatments like D1, D2 and S1 have a moderate effect on the biochemical parameters, as it is between the control and D1S1 treatments on the biplots and has medium-sized arrows pointing to it. The biochemical parameters that are most affected by the treatments are CAT, SOD, and H_2_O_2_, as they have the longest arrows and are aligned with the Dim1 axis, which explains most of the variance. The biochemical parameters that is least affected by the treatments include POD, having the shortest arrows and are perpendicular to the Dim1 axis, which explains less of the variance. The biochemical parameters like CAT and SOD are positively correlated, indicated by arrows pointing in the same direction. They are associated with oxidative stress and antioxidant activity in plants. In contrast, parameters like POD and RWC are negatively correlated, as indicated by arrows pointing in opposite directions. These parameters are associated with water status and peroxidase metabolism in plants.

### 3.6 Heatmap analysis

**Figure 6:**
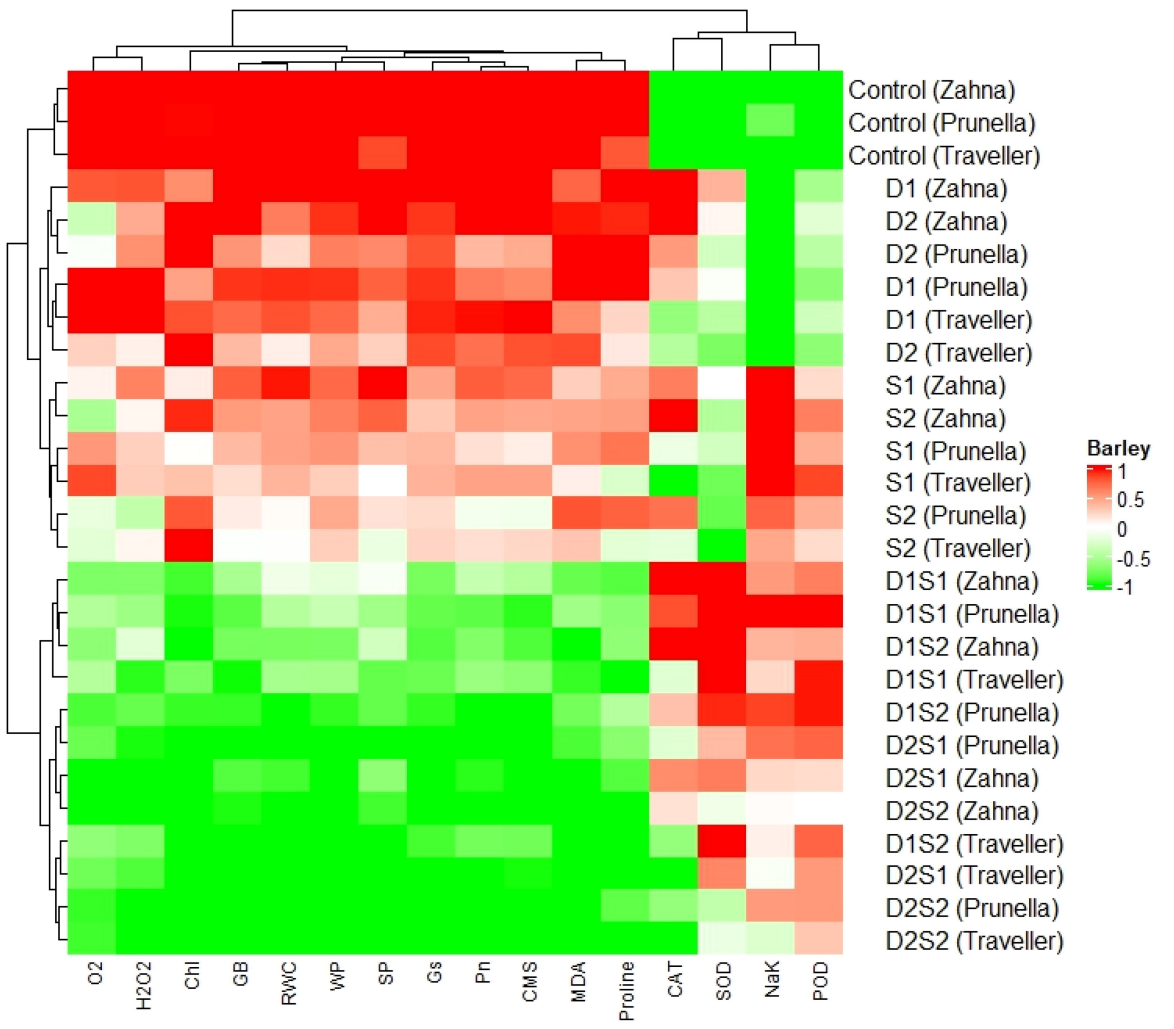
Cluster dendrogram heatmap illustrating the responses of physiological, biochemical traits to barley genotypes at individual and combined treatments of drought and salinity stresses.

The color gradient, ranging from red to green, signifies values from 1 to -1, respectively (**Figure 6**). Analysis of the heatmap reveals varying correlations between parameters and barley genotypes. For instance, O_2_^-^, and H_2_O_2_, exhibit positive correlations with D1S1 (Zahna), whereas SP and CMS show negative correlations with the same barley type.

Furthermore, distinct patterns of correlation emerge among different barley types. Control treatments (Zahna, Prunella, and Traveller) exhibit similar correlation patterns with most parameters, whereas D1S1 and D2S2 types demonstrate differing correlation patterns. The combination treatments revealed decreased values in most parameters compared with the sole treatments. However, different from other biochemical parameters, the antioxidants enzymes (especially POD), show higher expression in the salinity and combined treatments compared to the drought and control treatments.

## 4 Discussion

The present study investigated the responses of barley plants to salinity and drought stresses at physiological and biochemical levels, with a focus on understanding genotype-specific variations. The main research question addressed how barley copes with these stresses and how genetic factors influence its response mechanisms.

The physiological responses of barley genotypes to individual and combined drought and salinity treatments unveiled intriguing patterns. Notably, the genotype Zahna emerged as particularly resilient across various stress conditions, maintaining higher relative water content (RWC), water, and solute potentials compared to Traveller and Prunella (**Figures 1A–C**) This resilience was further underscored by Zahna’s ability to sustain higher chlorophyll levels and photosynthetic rates (Pn) under stress (**Figure 1D**), indicative of its superior stress tolerance mechanisms.

Chlorophyll level is a notable indicator of photosynthetic capacity (Kumar et al., 2022). Our results did not just reveal significant differences in chlorophyll content but also in photosynthetic rate and stomatal conductance among treatments and genotypes (**Figures 1E–G**). Salinity and drought stresses led to a marked reduction in chlorophyll levels across all genotypes, indicating stress-induced disruption of photosynthetic processes (Singh et al., 2021; Choudhary et al., 2023). Additionally, genotype-specific responses were evident, highlighting the role of genetic factors in shaping plant physiology (Alsamadany et al., 2023). Cell membrane stability showed a notable decrease under stress conditions compared to the control, suggesting stress-induced membrane damage and increased permeability (**Figure 1D**). As highlighted in previous studies (Qayyum et al., 2021; Choudhary et al., 2023), progressive declines in relative water content, water potential, and osmotic potential under stress conditions underscored the plants’ responses to water deficit and osmotic stress, with genotype-specific adjustments reflecting differences in water management strategies (**Figures 1A–C**).

Intricate modulation of antioxidant defense mechanisms and osmolyte accumulation was revealed in the biochemical responses of barley genotypes to stress. Notably, the genotype-specific variations in hydrogen peroxide (H_2_O_2_) levels underscored the differential regulation of oxidative stress responses (**Figure 2B**). Zahna exhibited lower superoxide (O_2_^-^) levels across most treatments, indicative of its enhanced capacity to scavenge reactive oxygen species (ROS). Maintaining a stable ROS concentration in abiotic stressed plants is often seen as beneficial for plant tolerance (Ouertani et al., 2022; Abdulbaki et al., 2024). Furthermore, the upregulation of antioxidant enzyme activities in response to stress highlights the pivotal role of enzymatic antioxidants in mitigating oxidative damage. Generally, Zahna’s better ability to modulate these antioxidant enzyme activities, such as SOD and CAT, highlights its potential for oxidative stress mitigation (**Figures 2E–H**). The present study is consistent with earlier results in barley, where analysis of antioxidant enzyme activities revealed genotype-specific responses to oxidative stress (Ouertani et al., 2022). Conversely, Prunella exhibited higher levels of malondialdehyde (MDA) concentration, suggesting greater susceptibility to oxidative damage, particularly under combined stress conditions (Singh and Rathore, 2018). The decline in proline and glycine betaine levels under stress conditions (**Figure 2G, H**) underscores their role as compatible solutes involved in osmotic adjustment and ROS scavenging (Olayinka et al., 2021).

In morphological parameters, considerable variations were observed across treatments and genotypes (**Table 2**). Incredibly, salinity treatments (S) unlike drought (D) tended to promote plant growth, as evidenced by increased plant height, leaf area, and number of leaves. This perceived improvement in growth in the barley genotypes could be attributed to better ion exclusion or osmotic adjustment (Hussain et al., 2020). However, the combined drought and salinity treatments induced significant variations in morphological traits, with genotype Zahna exhibiting taller plants and more leaves compared to Prunella and Traveller. (**Table 2**). This result further highlights the importance of understanding how treatments and genotype variations impact morphological traits, as shown in earlier reports (Shah et al., 2022; Mousa et al., 2024). Taller plants but lower leaf areas in Zahna may be seen as a compensatory growth survival strategy (Zhou et al., 2022) or could be associated with stress-induced ABA accumulation, which promotes cell expansion and elongation (Karlova et al., 2021). (**Table 2**). Interaction effects between treatments and genotypes highlighted the complexity of factors influencing plant growth and productivity, emphasizing the need for tailored management practices (**Table 2**).

The gene expression analysis provided valuable insights into the molecular mechanisms underlying stress responses in barley (**Figures 3A–G**). The upregulation of antioxidant-related genes, such as HvSOD, HvAPX, and HvCAT, under stress conditions, underscores their crucial role in mitigating oxidative stress. Zahna consistently demonstrated robust antioxidant defense mechanisms, especially under combined stress. Notably, the combination treatments involving moderate drought exhibited the highest expression of enzymatic antioxidant-related genes, highlighting the synergistic effects of drought and salinity stresses on gene regulation (Kamal et al., 2021). Nevertheless, the genotype-specific expression patterns of stress-responsive genes, such as HvHKT1;5, HvPRP1, HvDREB3, and HvDHN3, underscored the genetic diversity in stress tolerance mechanisms among barley genotypes (Ghomi et al., 2021). The genes, generally, exhibited intriguing patterns, revealing their potential importance in stress tolerance mechanisms (**Figure 3G**) Genes, like *OsMYB6* in rice and *HvPIP2;5* in barley have been identified to confer similar stress-responsive traits (Alavilli et al., 2016; Tang et al., 2019). These findings contribute to our understanding of the molecular basis of stress tolerance in barley.

The strong positive correlations among physiological traits such as RWC, WP, and SP suggest a coordinated response to maintain water status and solute balance, which are crucial under stress conditions (**Figures 4A, B**). These traits are central to the plant’s ability to uphold cellular integrity and function during adverse environmental conditions (Li et al., 2019). Conversely, the negative correlations observed between physiological parameters like NaK and CMS or Chl indicate a disruption in cellular and photosynthetic efficiency due to ionic imbalances and oxidative stress (Ran et al., 2022). This disruption can potentially lead to reduced plant growth and productivity, highlighting the challenges plants face in saline or drought conditions (Muhammad et al., 2021).

The proximity of Prunella and Traveller genotypes from the PCA biplots suggests similar biochemical adaptive strategies to stress, reflecting their genetic or phenotypic similarities (**Figures 5A, B**). In contrast, the genotype Zahna displays distinct biochemical responses with greater variability, revealing a more robust set of mechanisms to counteract stress, translating to higher stress resilience. Furthermore, the association of Traveller samples with ROS indicates a heightened oxidative stress response (Olayinka et al., 2021). In the same vein, the significant impact of the D1S1 treatment on biochemical parameters, particularly on antioxidants like CAT and SOD, is indicative of intense oxidative stress requiring active ROS-scavenging mechanisms (Melicher et al., 2022).

Interestingly, the heatmap analysis provides a nuanced view of genotype-specific responses, particularly under combination treatments (D1S1 and D2S2), which seem to exert more pronounced effects on biochemical parameters compared to single stress treatments (**Figure 6**). This suggests a non-linear response to combined stresses, where the interaction between different stress factors can exacerbate the stress response beyond what is observed under single stress conditions (Angon et al., 2022). Moreover, the increased expression of antioxidant enzymes, particularly POD, under salinity and combined treatments compared to drought and control conditions, further reflect the more severance of saline conditions on the growth and development of the genotypes, compared to the drought. The differential response of POD compared to other antioxidants like CAT and SOD might reflect its unique role or effectiveness in mitigating oxidative damage under salinity stress (Wang et al., 2022).

The interplay between drought and salinity stresses was most evident in the combined stress treatments, where Zahna shows a distinct advantage. The coordinated upregulation of antioxidant enzymes and stress-responsive genes in Zahna highlights a synergistic effect of drought and salinity on gene regulation. This genotype’s ability to modulate antioxidant defenses effectively mitigates oxidative damage, a critical factor for maintaining cellular homeostasis under combined stress. Conversely, genotypes Prunella and Traveller displayed less effective stress responses, with higher levels of oxidative damage markers such as MDA, particularly under combined stress conditions, indicating their relatively higher susceptibility to integrated stresses.

The present study mimics the natural conditions of both drought and salinity stresses occurring simultaneously, either due to the presence of common factors that can affect the plant at the same time or when plants exposed to drought stress led to an increase in soil salinity. Our findings further established the worsened effects of having the two stressors occurring at the same time since the integrated treatments have more negative consequences (**Figures 1**–**4**) (Angon et al., 2022). This tallies with previous studies reporting the severance of integrated stresses on plant’s growth. Our findings also agree with (Makhtoum et al., 2022) noting the more susceptibility of barley genotypes to salinity than drought.

## 5 Conclusion

In conclusion, this comprehensive study elucidates the multifaceted mechanisms underlying drought and salinity stress responses in barley, offering valuable insights into the adaptations employed by barley genotypes to cope with stress. Our study reveals the genotype-specific responses of barley to drought and salinity stresses, with Zahna emerging as a resilient cultivar characterized by superior physiological, biochemical, and morphological adaptations. Zahna maintains higher relative water content, chlorophyll levels, and photosynthetic rates under stress conditions, coupled with lower levels of oxidative stress markers and upregulated enzymatic antioxidant activities. Zahna displays favorable growth characteristics, indicating its potential as a stress-resilient cultivar. The gene expression analysis underscores the molecular mechanisms underlying stress tolerance in barley, laying the foundation for targeted breeding efforts aimed at enhancing stress resilience and ensuring food security in the face of climate change-induced abiotic stresses.

## Data availability statement

The original contributions presented in the study are included in the article/**Supplementary Material**. Further inquiries can be directed to the corresponding author.

## Author contributions

HA: Conceptualization, Writing – original draft, Writing – review & editing. AA: Methodology, Writing – original draft, Writing – review & editing. YA: Formal analysis, Data curation, Writing – original draft, Writing – review & editing.
