# Supplementary figures and images for: Unravelling drought and salinity stress responses in barley genotypes: physiological, biochemical, and molecular insights
Source: Front Plant Sci. 2024 Jul 10;15:1417021. doi: 10.3389/fpls.2024.1417021 (PMC11266107; doi:10.3389/fpls.2024.1417021)

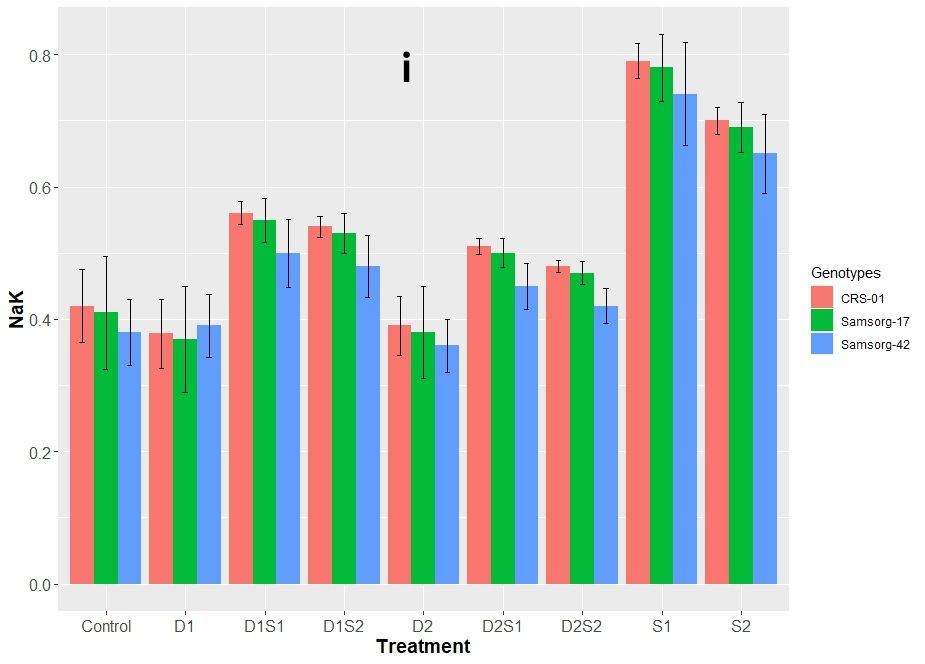

Supplement: Supplementary file 1 [file Image_1.jpeg]
