# Supplementary material for: Unravelling drought and salinity stress responses in barley genotypes: physiological, biochemical, and molecular insights
Source: Front Plant Sci. 2024 Jul 10;15:1417021. doi: 10.3389/fpls.2024.1417021 (PMC11266107; doi:10.3389/fpls.2024.1417021)
Supplement: Supplementary file 2 [file Table_1.docx]

|  | **SOD**  *Enzyme units* | **POD**  *Enzyme units* | **CAT**  *Enzyme units* | **Chl**  *(gKg^-1^)* | **CMS**  **%** | **H_2_O_2_**  *(AU μg^-1^ protein)* | **O_2_^-^**  *(AU μg^-1^ protein)* | **Proline**  *(μgg^-^ FW)* | **GB**  *(μgg^-^ FW)* | **MDA**  *.(nmolg^-1^FW)* | **Na^+^/K^+^** | **RWC**  % | **Ψ_w_ (-)**  (MPa) | **Ψ_s_**  (MPa) | **Pn** | **Gs** |
| --- | --- | --- | --- | --- | --- | --- | --- | --- | --- | --- | --- | --- | --- | --- | --- | --- |
| **Treatments (T)** | | | | | | | | | | | | | | |  |  |
| C | 17±0.7g | 0.22±0.02i | 6.0±0.2i | 1.70±0.02i | 70±3h | 2015±3i | 1450±4i | 21±0.7i | 60±3i | 8.0±0.5a | 0.37±0.02di | 78±2h | 0.42±0.02h | 0.85±0.02h | 38±2h | 780±4i |
| D1 | 28±0.5af | 0.45±0.03gh | 12±0.3h | 1.08±0.01h | 50±2g | 2450±2h | 1770±3h | 26±0.8h | 81±1h | 10±0.6b | 0.45±0.04gh | 55±1g | 0.60±0.02g | 0.97±0.01g | 30±1g | 600±5h |
| D2 | 30±0.6ae | 0.47±±0.03fg | 14±0.2g | 0.94±0.02g | 40±3e | 2475±3g | 1780±4g | 28±0.7g | 90±3g | 15±0.7f | 0.47±0.02g | 40±1c | 0.79±0.01f | 1.10±0.03f | 24±1e | 500±3g |
| S1 | 29±0.6a | 0.50±0.03f | 13±0.3f | 1.11±0.02f | 46±2f | 2500±3f | 1750±2f | 25±0.7f | 80±1f | 12±0.7e | 0.66±0.03bdf | 60±1f | 0.73±0.01e | 1.20±0.02e | 28±2f | 580±3f |
| S2 | 33±0.6d | 0.55±0.0e | 16±0.2e | 0.90±0.02e | 41±3e | 2550±3e | 1800±4e | 28±0.7e | 88±3e | 15±0.7d | 0.86±0.02e | 55±1e | 0.85±0.01d | 1.40±0.03d | 24±1e | 510±3e |
| D1S1 | 35±0.5d | 0.61±0.02d | 17±0.3d | 0.80±0.02d | 38±2d | 3000±4d | 2100±4d | 40±0.8d | 115±2d | 15±0.6a | 0.70±0.04d | 45±2d | 0.90±0.02c | 1.68±0.03c | 22±1d | 510±3d |
| D1S2 | 37±0.6c | 0.68±0.03c | 18±0.2c | 0.60±0.02c | 34±3c | 3050±3c | 2150±4c | 42±0.7c | 120±3c | 14±0.7c | 0..95±0.02c | 40±1c | 0.90±0.01c | 1.70±0.03c | 20±1c | 450±3c |
| D2S1 | 32±0.6b | 0.50±0.03b | 16±0.2b | 0.50±0.02b | 30±3b | 3100±3b | 2200±4b | 35±0.7b | 105±3b | 10±0.7b | 0.65±0.02b | 35±1b | 0.98±0.01b | 1.78±0.03b | 18±1b | 425±3b |
| D2S2 | 28±0.6a | 0.33±0.03a | 8±0.2a | 0.45±0.02a | 27±3a | 3150±3a | 2275±4a | 30±0.7a | 95±3a | 8±0.7a | 0.99±0.02a | 30±1a | 1.10±0.01a | 1.88±0.03a | 16±1a | 400±3a |
| LSD | 0.90 | 0.05 | 1.00 | 0.05 | 2.20 | 26.00 | 8.00 | 2.00 | 1.80 | 1.00 | 0.04 | 3.20 | 0.05 | 0.05 | 2.00 | 11.00 |
| **Genotypes (G)** | | | | | | | | | | | | | | |  |  |
| **Barley** | | | | | | | | | | | | | | |  |  |
| Traveller | 20±0.5d | 0.25±0.02f | 11±0.3f | 0.70±0.01f | 70±2f | 2000±4e | 1750±3f | 23±0.8f | 80±3df | 9±0.5ef | 0.50±0.05f | 60±3f | 0.58±0.02f | 0.73±0.02ef | 31±1e | 680±3f\ |
| Prunella | 23±0.6c | 0.26±0.02e | 13±0.4e | 0.81±0.02e | 72±1e | 2050±2d | 1725±4e | 26±0.8de | 82±2ce | 10±0.7e | 0.45±0.04ace | 63±2e | 0.50±0.01e | 0.75±0.02e | 32±1e | 670±3e |
| Zahna | 25±0.7b | 0.28±0.01d | 14±0.4d | 0.86±0.02d | 75±1d | 2025±4d | 1690±5d | 27±0.6cd | 85±5bd | 12.0±0.5acd | 0.40±0.03bd | 68±3cd | 0.40±0.01abd | 0.67±0.01bd | 34±2cd | 690±4d |
| **Significance** | | | | | | | | | | | | | | |  |  |
| G | * | ** | * | ** | * | ** | ** | ** | ** | ** | * | * | ** | ** | * | ** |
| T | * | * | * | ** | ** | * | ** | ** | ** | ** | ** | * | ** | ** | * | * |
| G x T | ** | * | * | * | ** | ** | ** | ** | * | ** | ** | * | ** | * | * | * |
